# Supplementary material for: Genome-Wide Association Study of Arabinoxylan Content from a 562 Hexaploid Wheat Collection
Source: Plants (Basel). 2023 Jan 1;12(1):184. doi: 10.3390/plants12010184 (PMC9823421; doi:10.3390/plants12010184)
Supplement: Supplementary file 1 [file plants-12-00184-s001.zip › GWAS-Arabonoxylan_Supplementary data-pr.pdf]

Supplementary information

Genome-wide association study of arabinoxylan content from a 562 hexaploid wheat collection

Myoung Hui Lee, Jinhee Park, Kyeong-Hoon Kim, Kyeong-Min Kim, Chon-Sik Kang, Go Eun Lee, Joon Yong Choi, Jiyoung Shon, Jong-Min Ko, and Changhyun Choi*

Wheat Research Team, National Institute of Crop Science, Rural Development Administration, Wanju, 55365, Republic of Korea

* Correspondence: chchhy@korea.kr; Tel.: +82-63-238-5454.

Suppementary Table S1. Wheat genotypes (n= 562) from 46 countries.

			Arabinoxylan (mg/g)		no.			Arabinoxylan (mg/g)	
no.	country	genotype	average	SD	no.	country	genotype	average	SD
1	Afghanistan	13	41.29	14.56	25	Macedonia	1	53.29	–
2	Argentina	5	44.98	12.84	26	Mexico	104	48.56	11.26
3	Australia	4	50.41	6.88	27	Mongolia	6	47.24	15.05
4	Austria	1	46.84	–	28	Nepal	2	55.95	2.26
5	Bosnia and Herzegovina	3	54.56	8.78	29	Netherlands	1	40.33	–
6	Brazil	1	41.82	–	30	Pakistan	3	37.59	12.49
7	Bulgaria	8	47.36	9.18	31	Poland	1	55.27	–
8	Canada	10	42.11	3.97	32	Portugal	8	46.84	5.32
9	China	31	35.88	18.31	33	Romania	2	48.26	3.97
10	Colombia	3	36.49	10.76	34	Russia	19	50.03	9.4
11	Croatia	4	48.74	13.74	35	Saudi Arabia	2	52.2	3.54
12	Egypt	3	54.53	3.065	36	South Africa	2	55.48	4.36
13	Ethiopia	22	45.75	9.46	37	Spain	6	50.12	9.77
14	Finland	1	30.11	–	38	Slovenia	1	51.43	–
15	France	4	47.01	17.59	39	Syria	2	45.49	4.78
16	Germany	1	52.82	–	40	Tajikistan	1	54.63	–
17	Greece	3	44.7	12.12	41	Tunisia	1	63.21	–
18	Hungary	6	51.78	3.12	42	Turkey	12	44.95	9.04
19	India	7	49.43	7.61	43	Ukraine	7	50.19	12.68
20	Japan	10	54.51	6.07	44	Uned States	47	47.49	7.695
21	Kyrgyzstan	1	24.18	–	45	Uzbekistan	2	52.68	4.2
22	Korea, North	4	49.12	8.43	46	Zimbabwe	1	49.60	–
23	Republic of Korea	127	56.91	7.88	47	unknown	57		
24	Lebanon	2	36.56	11.26					

SD, standard deviation.

Supplementary Table S2. Arabinoxylan distribution of three haplotypes in 562 wheat resources.

AX-94470319	AX-95086356	AX-94713015	AX-94534026	AX-95019636	AX-95092984	AX-94502724	AX-94934861	Average (mg/g)	SD (mg/g)
AA								55.08	9.44
GG								46.79	10.87
	AA							53.47	8.92
	GG							44.74	9.15
		AA						52.31	9.96
		GG						47.34	11.50
			CC					51.22	10.73
			TT					47.09	11.14
				CC				47.06	11.02
				TT				51.81	10.77
					AA			45.41	15.95
					CC			50.66	9.58
						CC		-	-
						TT		48.83	11.1
							AA	48.54	11.57
							GG	46.21	11.65
AA	AA	AA						57.17	6.08
AA	AA	GG						55.65	7.12
AA	GG	AA						53.75	9.87
AA	GG	GG						43.95	8.72
GG	AA	AA						51.78	8.69
GG	AA	GG						48.97	9.67
GG	GG	AA						43.22	9.49
GG	GG	GG						45.34	8.78

SD, standard deviation.

Supplementary Table S3. Arabinoxylan content of three allele combinations in 41 Republic of Korea wheat cultivars.

AX-94470319	AX-95086356	AX-94713015	AX-94534026	AX-95019636	Average (mg/g)	SD (mg/g)
AA					58.11	2.20
GG					58.09	5.67
	AA				58.10	5.37
	GG				-	-
		AA			58.69	6.24
		GG			57.41	4.20
			CC		–	-
			TT		58.10	5.64
				CC	57.63	6.23
				TT	58.46	4.56
AA	AA	AA			58.39	6.21
AA	AA	GG			57.64	3.26
GG	AA	AA			59.11	6.63
GG	AA	GG			57.25	4.93

SD, standard deviation.

Supplementary Table S4. Arabinoxylan content of 41 Republic of Korean wheat cultivars. SD, standard deviation.

No.	Cultivar	Arabinoxylan (mg/g)		No.	Cultivar	Arabinoxylan (mg/g)	
		Average	SD			Average	SD
1	Milsung	45.10	2.07	22	Younbaek	51.04	2.04
2	Saeol	52.63	1.83	23	Olgeru	54.98	3.46
3	Shinmichal	60.65	2.01	24	Uri	58.90	2.36
4	Ol	65.43	2.52	25	Jeokjoong	60.82	4.33
5	Hanbaek	67.39	6.06	26	Cheonggye	60.53	1.17
6	Baekchal	55.76	1.75	27	Geuru	53.59	3.02
7	Sukang	62.56	5.86	28	Dajoong	61.51	4.85
8	O-free	59.43	3.24	29	Saekeumkang	57.77	5.22
9	Jonong	55.62	2.73	30	Jopoom	54.87	0.60
10	Arijinheuk	62.44	5.49	31	Gobun	50.84	3.68
11	Jojoong	66.66	4.89	32	Shinmichal1	65.72	5.09
12	Taejoong	56.19	4.65	33	Eunpa	67.58	6.49
13	Hojoong	57.09	2.85	34	Joa	50.98	4.43
14	Keumkang	44.11	4.48	35	Jinpoom	54.53	3.02
15	Suan	58.34	3.23	36	Alchan	60.36	4.61
16	Wooju	55.22	4.39	37	Joeun	61.84	1.94
17	Jokyung	54.66	2.32	38	Johan	60.19	2.41
18	Hwanggeumal	58.16	6.92	39	Joongmo2008	58.22	3.91
19	Namhae	61.16	4.00	40	Joongmo2015	57.23	3.13
20	Dabun	56.60	2.82	41	Tapdong	61.38	2.85
21	Baekjoong	63.89	3.46				

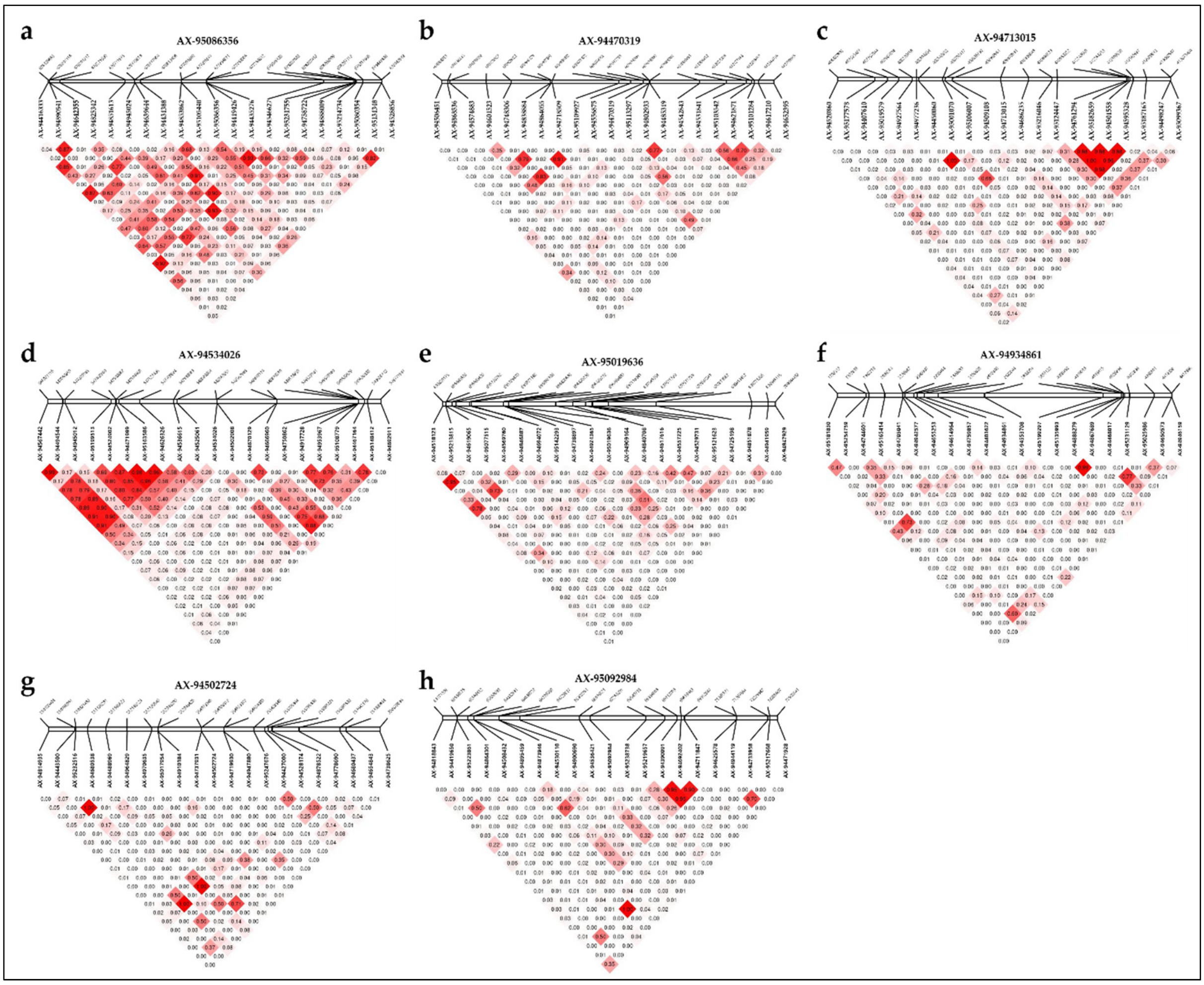

Supplementary Figure S1. Linkage disequilibrium (LD) patterns among 20 simulated SNPs based on r^2 using confidence intervals. **(a)** AX-95086356 on chromosome 1B, **(b)** AX-94470319 on chromosome 4B, **(c)** AX-94713015 on chromosome 5D, **(d)** AX-9454026 on chromosome 5D, **(e)** AX-95019636 on chromosome 5B, **(f)** AX-94934861 on chromosome 7D, **(g)** AX-94502724 on chromosome 3D, and **(h)** AX-95092984 on chromosome 7B. Red blocks denote high LD.

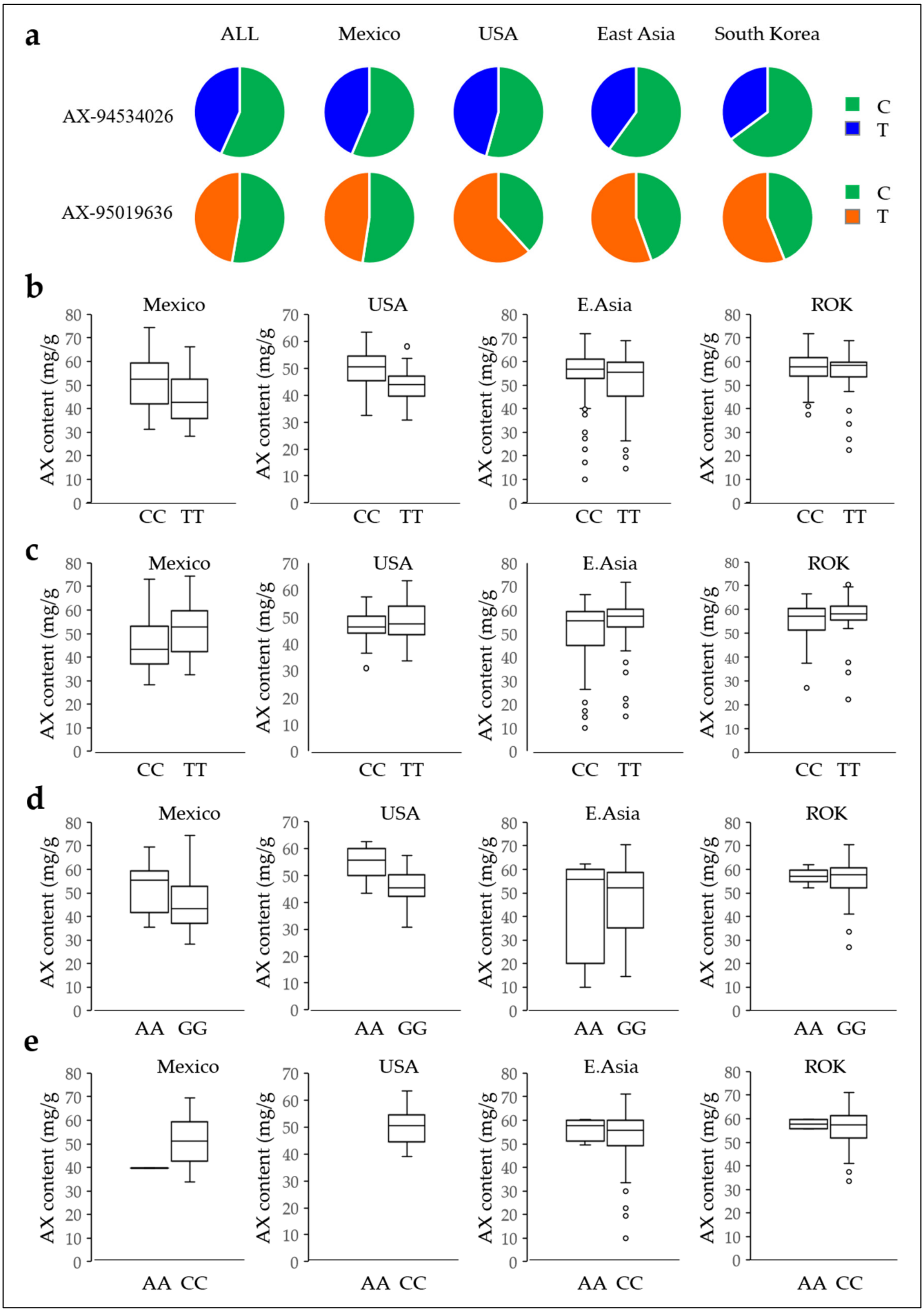

Supplementary Figure S2. Arabinoxylan level by country according to the two SNP alleles. Cysteine and thymine rates by country (**a**); the AX level by country according to SNP allele in AX-94534026 (**b**), AX-95019636 (**c**), AX-94934861 **(d)**, and AX-95092984 **(e)**. The X-axis displays the two alleles, and the Y-axis represents the AX content. AX, arabinoxylan; C, cytosine; T, thymine. ROK, republic of Korea.

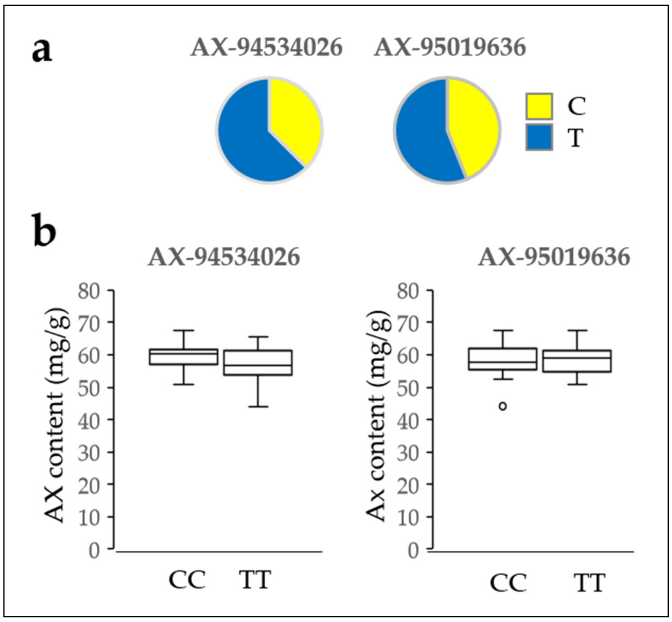

Supplementary Figure S3. Arabinoxylan content of 41 Korean varieties in AX-94534026 and AX-95019636 SNPs. The adenine and guanine allele rates (**a**) and the AX content (**b**) in alleles of AX-94534026 and AX-95019636. AX, arabinoxylan; C, cytosine T, thymine.
